# Creation, characterization, and assignment of opsonic values for a new pneumococcal OPA calibration serum panel (Ewha QC sera panel A) for 13 serotypes: Erratum

**DOI:** 10.1097/MD.0000000000011171

**Published:** 2018-06-18

In the article, “Creation, characterization, and assignment of opsonic values for a new pneumococcal OPA calibration serum panel (Ewha QC sera panel A) for 13 serotypes”,^[1]^ which appeared in Volume 97, Issue 17 of *Medicine*, the descriptions of Tables 6 and 7 should state that the bold text in the tables indicates that at least 1 laboratory reported an irregular result for that sample for that serotype in at least 1 run.
